# Selective Fluorescence Detection of Glyphosate Pesticide Residue Based on Fe^3+^ Modulated SiQDs Nanosensors

**DOI:** 10.3390/s26092851

**Published:** 2026-05-02

**Authors:** Ruonan Li, Jian Xu, Fankui Zeng

**Affiliations:** 1 Research Center for Natural Medicine and Chemical Metrology, Lanzhou Institute of Chemical Physics, Chinese Academy of Sciences, Lanzhou 730000, China; liruonan@licp.cas.cn; 2 University of Chinese Academy of Sciences, Beijing 100049, China

**Keywords:** SiQDs, glyphosate pesticide, Fe^3+^ modulated, fluorescence detection, nanosen-sors, sample testing

## Abstract

In this paper, SiQDs were synthesized using 3-aminopropyltrimethoxysilane, an organosilicon source, via the room temperature stirring method under atmospheric pressure. Based on the “Turn-off” and “Turn-on” fluorescence response mechanisms, the SiQDs/Fe^3+^ fluorescent probe was constructed to quantitatively detect glyphosate according to the interaction between Fe^3+^ and glyphosate. Subsequently, the impacts of pH, incubation temperature, and reaction time on the detection of glyphosate were systematically investigated. Under the optimized detection parameters, the fluorescent probe exhibited a linear range of 2–10 μg/mL and a detection limit of 394.74 ng/mL. The constructed fluorescent probe demonstrated outstanding anti-interference performance. It was applied to actual samples of potato and yam, yielding satisfactory detection results with recovery values between 91.69% and 104.53%. These findings provide novel ideas and theoretical support for glyphosate residue detection.

## 1. Introduction

As one of the most widely used organophosphorus herbicides in the world, glyphosate has the significant advantages of a broad-spectrum weeding effect, strong conductivity, high efficiency, low cost and low toxicity. Glyphosate blocks the shikimic acid metabolic pathway mainly by inhibiting enzyme activity, which in turn inhibits protein production [1]. Since this enzyme does not exist in animals, glyphosate has low toxicity to mammals, which is one of the major reasons for its widespread use.

Since its introduction in the 1970s, the annual global production of glyphosate has risen steadily, and it is widely used to control different types of weeds in more than 100 crops in 130 countries and territories [2,3]. Such high-dose use has resulted in widespread dispersion of glyphosate into the environment through runoff and the atmospheric water cycle. Although degradation of glyphosate can occur in soil, water, and plants, the stable C–P chemical bonds in the molecule protect it from complete degradation [4]. Therefore, glyphosate is a potential threat to biota and humans. Studies have shown a correlation between glyphosate residues and cancer, respiratory diseases, DNA damage, and neurological disorders [5,6,7]. As the shikimic acid metabolic pathway of mangiferic acid is also present in some fungi and bacteria [8,9], low doses of glyphosate can also interfere with the composition of the gut microbiota and modulate the neuroimmune-endocrine system leading to intestinal dysbiosis [10]; Lesseur examined the levels of glyphosate and its degradation product, aminomethylphosphonic acid (AMPA), in urine samples from 163 pregnant women in mid-pregnancy, to study the association between prenatal glyphosate exposure and the duration of pregnancy, and they found that when the general population is widely exposed to glyphosate, it may affect reproductive health by shortening the duration of pregnancy [11]. As a result, the World Health Organization (WHO) has set a maximum contaminant level of glyphosate residue in drinking water at 5.325 µM (900 µg/L). Therefore, the quantitative detection of glyphosate residues in agricultural products is particularly important.

With the rapid development of science and technology, glyphosate detection technology has gradually evolved from a single, inefficient method in the early stage to diversification, high efficiency and precision. Early glyphosate detection relied on technologies such as HPLC and GC-MS, which can achieve trace detection but require complex pre-treatment, and the detection cycle can take hours or even days, making it difficult to meet the needs of on-site screening [12,13,14]. Although immunoassay (e.g., ELISA) is easy to operate, antibody specificity dictates its susceptibility to matrix interference, and the method has a risk of false positives [15,16,17]. In addition, the high-water solubility and poor stability of glyphosate further increase the difficulty of detection, and with the breakthroughs in nanomaterials and intelligent sensing technology, fluorescent quantum dot-based sensing platforms for pesticide residue monitoring have attracted extensive research interest.

**Figure 1:**
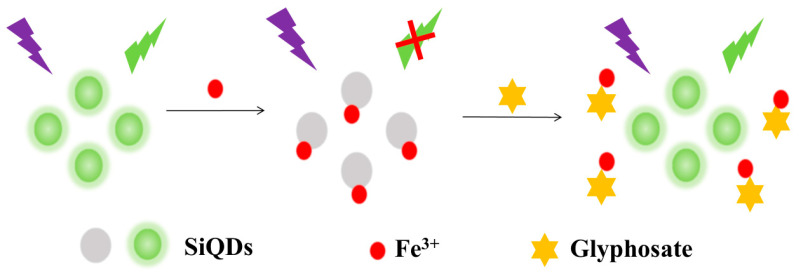
Schematic diagram of SiQDs/Fe^3+^ for the detection of glyphosate.

Therefore, in this paper, green-emitting SiQDs were prepared by a simple room temperature stirring reaction—an approach featuring mild reaction conditions, straightforward operation and no complex equipment requirements. Based on the fluorescence “Turn-off” and “Turn-on” switching mechanisms of SiQDs, glyphosate residues were quantitatively determined. The proposed experimental strategy is shown in Figure 1. Fe^3+^ is susceptible to ligand interaction with functional groups such as -OH and -COOH on the surface of SiQDs, resulting in static fluorescence quenching of SiQDs [18,19]. When glyphosate was added to the system, the phosphinic acid (-PO_3_H_2_) and carboxyl group (-COOH) in its molecular structure could complex with Fe^3+^, thus restoring the fluorescence signal of SiQDs [20,21]. Notably, this Fe^3+^-mediated fluorescent sensing platform has been successfully applied to the actual sample analysis of glyphosate residues, verifying its excellent practicability and potential for on-site rapid detection in complex real matrices.

## 2. Experimental Section

### 2.1. Materials and Instruments

3-aminopropyltrimethoxysilane (APTMS) was purchased from Macklin Biochemical Co., Ltd. (Shanghai, China). Glyphosate, parathion-methyl, methamidophos, malathion, and chlormequat were supplied by the J&K Scientific Company (Beijing, China). Sodium ascorbate (SA) was purchased from Aladdin Biochemical Technology Co., Ltd. (Shanghai, China). Glucose, xylose, lactose, and sodium acetate were purchased from Kermel Chemical Reagent Co., Ltd. (Tianjin, China). All reagents were of analytical grade and used without further purification.

Transmission electron microscope (TEM, JEM-2010, JEOL Ltd., Tokyo, Japan). Fourier transform infrared spectrometer (FTIR, Bruker V70, Bruker Corporation, Berlin, Germany). X-ray photoelectron spectrometer (XPS, ESCALAB 250Xi, ThermoFisher Scientific, Waltham, MA, USA). Fluorescence spectrophotometer (Fluoromax 4, HORIBA Scientific, Kyoto, Japan). UV-visible spectrophotometer (Lambda750, PerkinElmer Inc., Buckinghamshire, UK). High-resolution X-ray diffractometer (XRD, D8Discover25, Bruker Corporation, Germany).

### 2.2. Synthesis of SiQDs

SiQDs were synthesized with reference to the existing literature, on the basis of which they were modified appropriately [22]. Briefly, 2.00 mL of SA (100 mM) with 1.60 mL of APTMS (840 mM) was added to 6.4 mL of ultrapure water, and then the mixed solution was immediately transferred to a thick-walled pressure-resistant tube. The reaction was carried out for 1 h at room temperature (25 °C) under normal pressure. Subsequently, the prepared SiQDs were dialyzed in ultrapure water for 8 h to eliminate residual unreacted precursors, and the resulting SiQDs solution was stored at 4 °C for further characterization and assay.

The quantum yields of SiQDs were calculated according to the literature methods [23,24]. Using quinine sulfate as a reference solution (fluorescence quantum efficiency of 54%), the quantum yield of SiQDs was calculated by the following equation (Equation (1)).

(1) $$\Phi_{S}=\Phi_{R}\frac{I_{S}A_{R}\eta_{S}^{2}}{I_{R}A_{S}\eta_{R}^{2}}$$

The subscripts *S* and *R* in the formula represent the sample solution and reference solution, respectively. The symbols *Ф*, *I*, *A* and *η* correspond to the fluorescence quantum yield, absorbance, integrated area of the fluorescence spectrum and refractive index, in that order.

### 2.3. Detection of Fe^3+^ Based on the SiQDs

In a series of 5 mL clean centrifuge tubes, 30 μL of SiQDs solution with ultrapure water was added to each centrifuge tube, and then different volumes of Fe^3+^ solution (1 mM) were added to each centrifuge tube separately to reach a final volume of 4 mL. Subsequently, it was transferred to a vortex mixer for mixing, and the reaction was carried out for a period of time at a certain temperature. The fluorescence spectra of the mixed solutions were measured. The ratio of fluorescence intensity of SiQDs before and after the addition of Fe^3+^ was expressed as F/F_0_. (F and F_0_ are the fluorescence intensities of SiQDs at 500 nm in the presence and absence of Fe^3+^, respectively.)

### 2.4. Detection of Glyphosate by SiQDs/Fe^3+^ Fluorescent Probe

In a series of 5 mL clean centrifuge tubes, 30 μL of SiQDs solution with ultrapure water was added to each centrifuge tube, 160 μL of Fe^3+^ solution (1 mM) was added to each centrifuge tube, and then transferred to a vortex mixer for mixing, and the reaction was carried out for 10 min at a room temperature of 25 °C. Then, different volumes of glyphosate solution were added to each centrifuge tube. Then, different volumes of glyphosate solution were added to each centrifuge tube, which were incubated at a certain temperature to fully react. The intensity of the fluorescence emission peak at 500 nm was measured. The ratio of fluorescence intensity of the SiQDs/Fe^3+^ system before and after the addition of glyphosate was expressed as F/F_0_. (F and F_0_ are the fluorescence intensity of SiQDs at 500 nm in the presence and absence of glyphosate, respectively.)

Subsequently, in order to further reduce the concentration of the detection limit, the fluorescence spectra of the mixed solutions were re-measured after diluting the concentration of SiQDs by 3-fold and Fe^3+^ by 10-fold following the above procedure.

### 2.5. Detection of Glyphosate in Real Samples

The reliability of the assay system was verified by determining the recovery of glyphosate in potato and yam samples. Potatoes and yams were purchased from local markets (Lanzhou, China). The following procedure was followed with slight modifications according to the literature [25]. Firstly, 1 g of potato and yam samples were accurately weighed, 15 mL of acetonitrile was added for extraction and homogenized in an ultrasonic water bath. The mixed solutions were then centrifuged at 10,000 rpm for 10 min, and the supernatant was collected and filtered using a 0.22 µm microporous membrane. Then, the glyphosate standard solution was added to the actual potato and yam samples so that the final concentrations of glyphosate in the actual samples were 6 μg/mL, 8 μg/mL, and 10 μg/mL, respectively. Finally, the fluorescence emission intensities of the SiQDs at 500 nm were measured according to the optimal conditions obtained from the experimental results.

## 3. Results and Discussion

### 3.1. Characterization of SiQDs

**Figure 2:**
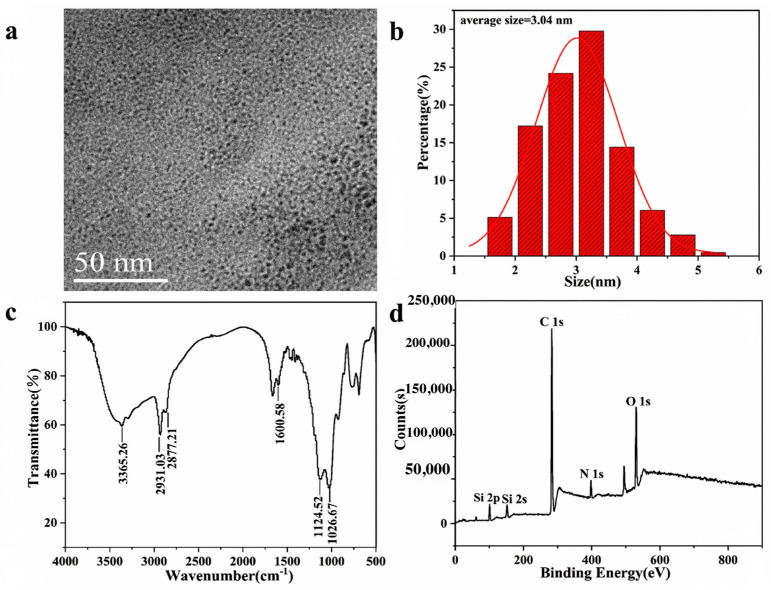
(**a**) The TEM image of the SiQDs; (**b**) The particle size distribution of the SiQDs; (**c**) The FTIR spectrum of the SiQDs; (**d**) The XPS spectra of SiQDs.

Firstly, the prepared SiQDs were characterized using TEM, and the relevant morphological features are shown in Figure 2a, revealing that the SiQDs are in the form of spherical particles. Figure 2b presents the particle size distribution analyzed from the TEM image of SiQDs through Nano measure software (Nano Measurer 1.2.5), and their sizes were mainly distributed between the range of 1.5–5.0 nm with an average size of 3.04 nm.

In order to determine the major chemical bonds of SiQDs, FTIR spectroscopy was used to unfold the analysis. As shown in Figure 2c, the 3365.26 cm^−1^ absorption peak originates from the stretching vibration of the O-H bond, and the absorption peaks at 2931.03 cm^−1^ and 2877.21 cm^−1^ are both attributed to the stretching vibration of the C-H bond. The absorption peak at 1600.58 cm^−1^ corresponds to the bending vibration of the N-H bond. The absorption peak at 1124.52 cm^−1^ and the absorption peak at 1026.67 cm^−1^ are both caused by the stretching vibration of Si-O bonds [26]. The presence of these chemical bonds indicates the successful synthesis of SiQDs and the presence of abundant functional groups, such as amino and hydroxyl groups, on the surface. Subsequently, XPS analysis was carried out to investigate the surface elements and chemical states of SiQDs, and the full XPS spectrum of SiQDs showed four typical peaks of Si 2p, C 1s, N 1s and O 1s (Figure 2d). The atomic contents of Si, C, N, and O in SiQDs were 6.34%, 73.12%, 5.05%, and 15.49%, respectively, by semi-quantitative XPS analysis.

**Figure 3:**
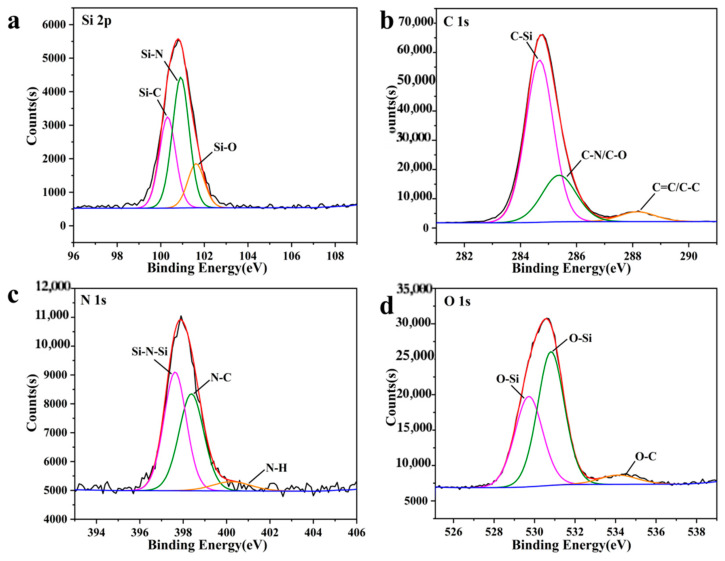
High resolution XPS image of (**a**) Si 2p; (**b**) C 1s; (**c**) N 1s; (**d**) O 1s.

Then, a split-peak fit was performed for each of the four typical peaks. The Si 2p fine spectra showed the presence of three silicon atoms corresponding to Si-C at 100.31 eV, Si-N at 100.92 eV, and Si-O groups at 101.62 eV (Figure 3a), which is in agreement with that shown by the data obtained by FTIR. The C 1s fine spectra showed the presence of three species belonging to Si -C, C-O/C-N, and C=C/C-C (Figure 3b). The N 1s fine spectrum consists of three peaks with binding energies of 397.33 eV, 398.08 eV, and 407.29 eV, respectively, which are attributed to Si-N-Si, N-C, and N-H groups (Figure 3c). The O 1s fine spectrum shows the presence of two O-Si groups at the binding energies of 529.72 eV and 530.81 eV, the presence of two O-Si groups at a binding energy of 529.72 eV and 530.81 eV, and the presence of O-C groups at a binding energy of 534.14 eV (Figure 3d).

**Figure 4:**
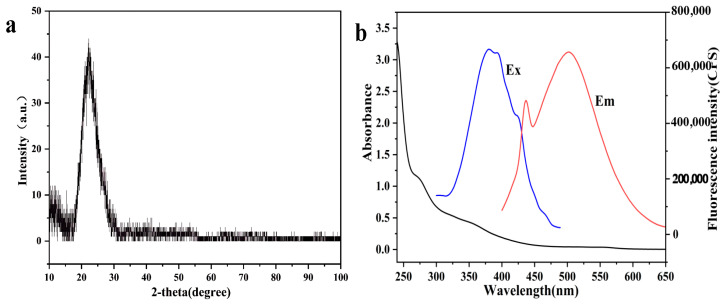
(**a**) The XRD image of the SiQDs. (**b**) UV-Vis absorption spectrum (black curve), fluorescence excitation spectrum (blue curve) and emission spectrum (red curve) of SiQDs.

Finally, the SiQDs were irradiated by an X-ray beam, and their diffraction patterns were resolved in order to obtain key information on the chemical composition, structure of the molecules, and microscopic morphology of the SiQDs. The XRD patterns of the SiQDs showed a broad diffraction peak centered at 2θ = 22.23° (JCPDS 29-0085). This result indicates that the SiQDs are in an amorphous state (Figure 4a).

The optical properties of SiQDs were further investigated by fluorescence spectra and UV-vis absorption spectra, and the results obtained are shown in Figure 4b. The fluorescence emission spectrum of SiQDs showed the strongest peak at 500 nm (red line) when 380 nm was used as the fluorescence excitation wavelength. The fluorescence quantum yield of SiQDs was 13.97%, calculated by Equation (1) of 2.2 using quinine sulfate as the reference solution.

### 3.2. Detection of Fe^3+^ Based on the “Turn-Off” Fluorescence Mechanism of SiQDs

**Figure 5:**
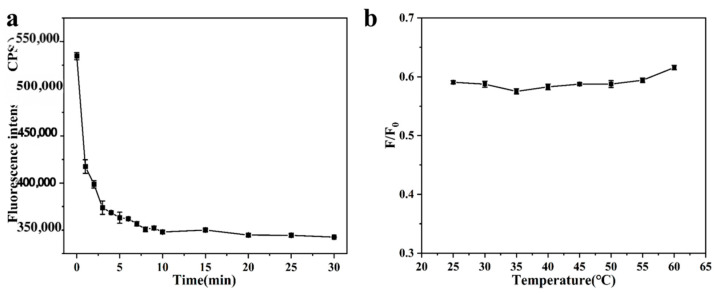
(**a**) Effect of reaction time on the fluorescence intensity of SiQDs; (**b**) Effect of incubation temperatures on the fluorescence intensity ratio (F/F_0_) of SiQDs.

The experimental conditions for the detection of Fe^3+^ by SiQDs were initially investigated and optimized. The influences of incubation time and temperature on the fluorescence intensity of the detection system were investigated. From Figure 5a, it can be found that the fluorescence intensity of the detection system gradually decreased with prolonged incubation time until it stabilized after 10 min. Figure 5b shows that the variation in the change in fluorescence intensity ratio F/F_0_ was not significant as the reaction temperature was increased from 25 °C to 60 °C. Therefore, the incubation time was chosen to be 10 min, and the incubation temperature was chosen to be 25 °C for the subsequent experiments.

**Figure 6:**
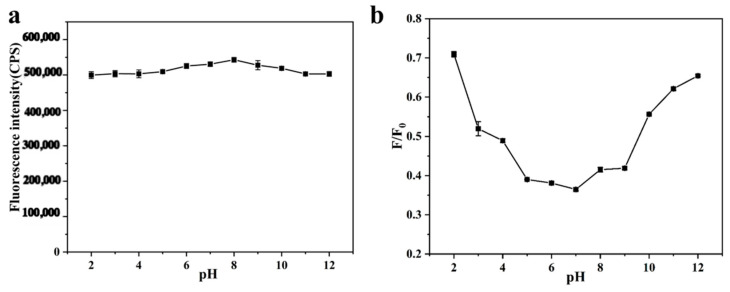
(**a**) Effect of pH on the fluorescence intensity of SiQDs; (**b**) Effect of pH on the fluorescence intensity ratio (F/F_0_) of the detection system.

Next, in order to determine the effect of the pH of the detection system on Fe^3+^, the changes in the fluorescence intensity of the SiQDs alone as well as the detection system at different pHs were investigated. From Figure 6a, it can be found that the effect of pH change on SiQDs itself is very small or even negligible; from Figure 6b, it can be seen that when the pH of the detection system is increased from 2 to 7, the fluorescence intensity ratio gradually decreases, which indicates that the burst effect of Fe^3+^ is gradually strengthened. Conversely, when the pH of the detection system is increased from 7 to 12, the fluorescence intensity ratio F/F_0_ gradually increases, and the burst effect of Fe^3+^ is gradually weakened, so the final choice was to set the pH of the detection system to seven. After that, the following experiments were carried out under these optimized experimental conditions.

**Figure 7:**
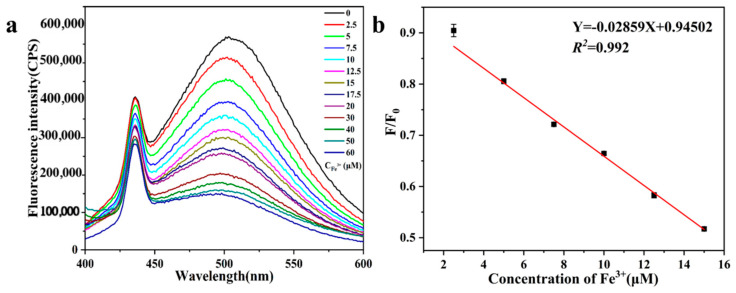
(**a**) Fluorescence intensity at different Fe^3+^ concentrations; (**b**) Linear relationship between fluorescence intensity ratio (F/F_0_) and Fe^3+^ concentration (2.5–15 μM).

From the fluorescence spectra Figure 7a, it can be seen that upon adding different concentrations of Fe^3+^ standard solution to the aqueous solution of SiQDs, the fluorescence intensity of the solution at 500 nm decreased gradually with the increase in Fe^3+^ concentration, and the Fe^3+^ concentration exhibited a good linear relationship in the range of 2.5–15 μM, and the linear equation is Y = −0.0286X + 0.945 (R^2^ = 0.992), and the limit of detection (LOD) was calculated to be 1.27 μM (Figure 7b).

**Figure 8:**
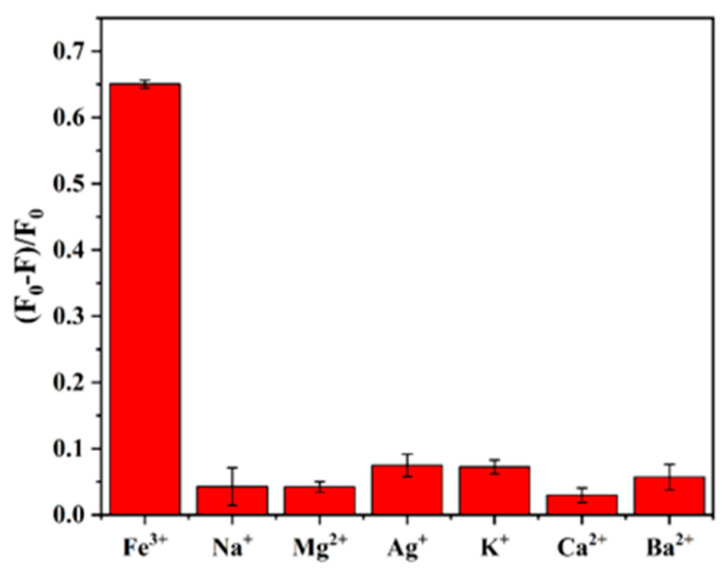
Fluorescence bursting efficiency of SiQDs induced by Fe^3+^ and other interfering ions ((F_0_ − F)/F_0_).

In addition, 200 μM of potential interfering substances (KCl, NaCl, CaCl_2_, BaCl_2_, MgCl_2_, AgNO_3_) were analyzed and detected to evaluate the selectivity of the fluorescence detection system at a Fe^3+^ concentration of 40 μM. As shown in Figure 8, under the same experimental conditions, the fluorescence bursting efficiency (F_0_-F)/F_0_ of only Fe^3+^ was much higher than that of other cations. The above results demonstrated the good stability and anti-interference ability of the detection system.

### 3.3. SiQDs/Fe^3+^ Fluorescent Probe “Turn-On” Mechanism for Glyphosate Detection

Previous studies have demonstrated a strong coordination interaction between glyphosate and Fe^3+^ [27]. Based on this finding, we postulated that the SiQDs/Fe^3+^ system was expected to be an effective solution for the detection of glyphosate. In order to verify the expected results of the SiQDs/Fe^3+^ system for the detection of glyphosate, the interaction between glyphosate and the SiQDs/Fe^3+^ system was further investigated.

**Figure 9:**
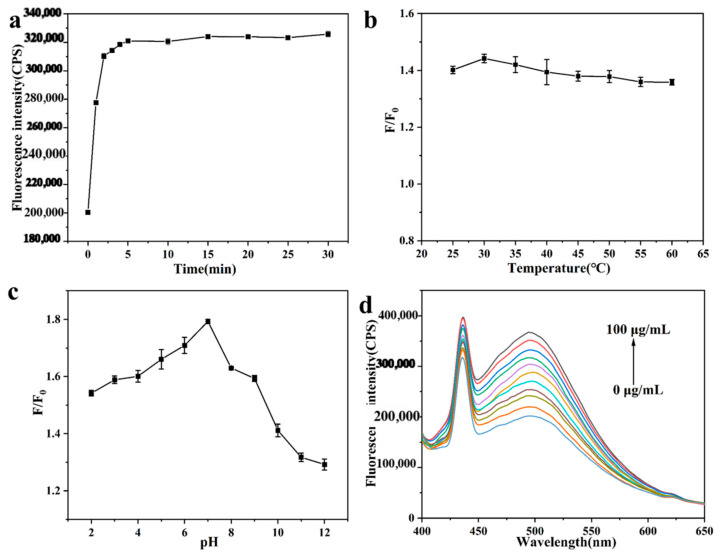
Effect of (**a**) SiQDs-Fe^3+^ reaction time; (**b**) Fe^3+^ and glyphosate incubation temperature; (**c**) pH on the detection of glyphosate; (**d**) Fluorescence spectra of SiQDs/Fe^3+^ fluorescent probes at different concentrations of glyphosate.

Usually, the kinetic reaction mechanism is determined by the incubation temperature together with the reaction time. Therefore, we first optimized the incubation time of glyphosate with the SiQDs/Fe^3+^ system. From Figure 9a, it was found that the fluorescence intensity of the detection system increased with the increase in reaction time and peaked at 5 min, and then kept the fluorescence intensity constant. After considering various factors, 5 min was chosen as the optimal reaction time for glyphosate.

Next, the influence of incubation temperature on the fluorescence intensity ratio F/F_0_ of the detection system was investigated. Figure 9b shows that the fluorescence intensity ratio F/F_0_ increases and then decreases as the reaction temperature increases from 25 °C to 60 °C, and reaches the maximum value at 30 °C, so the incubation temperature was chosen to be 30 °C for subsequent experiments.

The pH of the glyphosate assay system is an important factor affecting the experimental results. Subsequently, the effect of pH of the assay system on glyphosate activity was investigated. From Figure 9c, when the pH of the detection system was increased from 2 to 7, the fluorescence intensity ratio F/F_0_ gradually increased, indicating that the fluorescence intensity recovery effect of the system was gradually increased by glyphosate, whereas when the pH of the detection system was increased from 7 to 12, the fluorescence intensity ratio F/F_0_ gradually decreased, and the fluorescence intensity recovery effect was gradually reduced, so the final choice was to set the pH of the detection system to seven for the subsequent experiments.

**Figure 10:**
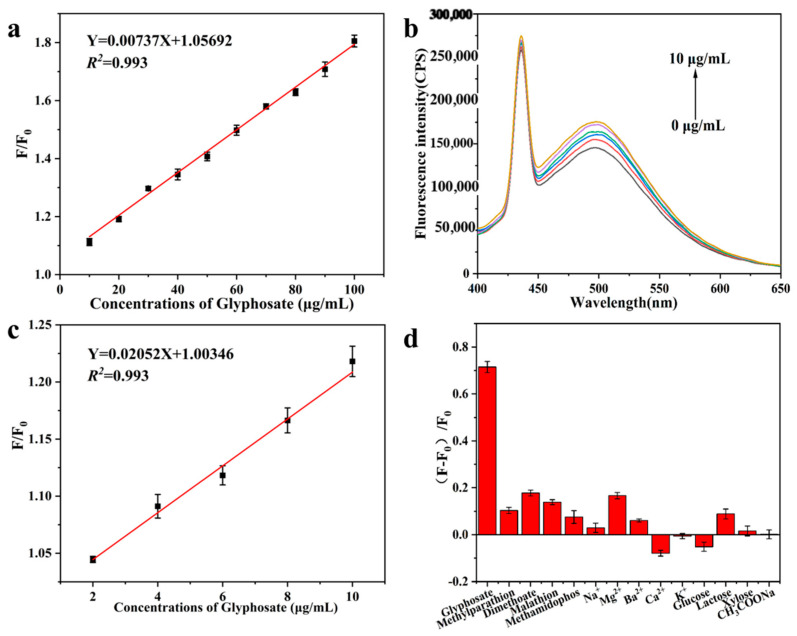
(**a**) Linear relationship between fluorescence intensity ratio (F/F_0_) and glyphosate concentration (10–100 μg/mL); (**b**) Fluorescence spectra of SiQDs/Fe^3+^ fluorescent probes at different low concentrations of glyphosate; (**c**) Linear relationship between fluorescence intensity ratio (F/F_0_) and low concentration of glyphosate (2–10 μg/mL); (**d**) Fluorescence recovery efficiency ((F − F_0_)/F_0_) between glyphosate and other interferences induced SiQDs/Fe^3+^.

Under the optimal experimental conditions, various concentrations of glyphosate standard solution were added to the SiQDs/Fe^3+^ system, and the fluorescence changes in the detection system are shown in Figure 9d. As can be seen from the fluorescence spectrogram, the fluorescence intensity of the solution at 500 nm showed an increasing trend with the increase in glyphosate concentration. From Figure 10a, it can be seen that at a Fe^3+^ concentration of 40 μM and when the glyphosate concentration was in the range of 10–100 μg/mL, the fluorescence intensity ratio, F/F_0_, increased linearly with the glyphosate concentration, and there was a good linear relationship of Y = 0.00737X + 1.057 (*R^2^* = 0.993), and the LOD was calculated to be 4.96 μg /mL.

In order to further lower the detection limit and improve the sensitivity of the assay. In the subsequent experiments, the fluorescence spectra of the mixed solution were measured again after reducing the SiQDs concentration by three times and the concentration of Fe^3+^ by 10 times in the follow-up experiments. As can be seen in Figure 10b, the fluorescence intensity of the detection system at 500 nm gradually increased with the increase in the low concentration of glyphosate, exhibiting a similar trend to that observed with high concentrations of glyphosate. When the glyphosate concentration was in the range of 2–10 μg/mL, the fluorescence intensity ratio F/F_0_ increased with the increase in glyphosate concentration, and there was a good linear relationship between them, Y = 0.0205X + 1.00346 (*R^2^* = 0.993), and the LOD was calculated to be 394.74 ng/mL (Figure 10c).

As a crucial indicator of a reliable fluorescent probe detection system, high selectivity is essential for accurate target analysis. In this chapter, the specific response of the detection system to the target analytes was further investigated by interference experiments. Figure 10d shows the change in fluorescence recovery efficiency (F-F_0_)/F_0_ in the presence of 50 μg/mL of other pesticides (methyl parathion, parathion, malathion, methamidophos) and 200 μM of potential environmental interferents (KCl, NaCl, CaCl_2_, BaCl_2_, MgCl_2_, glucose, xylose, lactose, sodium acetate). Upon excitation at 380 nm, only glyphosate induced obvious fluorescence enhancement at 500 nm, while other interfering pesticides and substances caused only relatively small or negligible changes in fluorescence intensity, indicating that the fluorescent probe detection system possesses excellent selectivity.

### 3.4. Mechanistic Study of Glyphosate Detection by SiQDs/Fe^3+^ System

**Figure 11:**
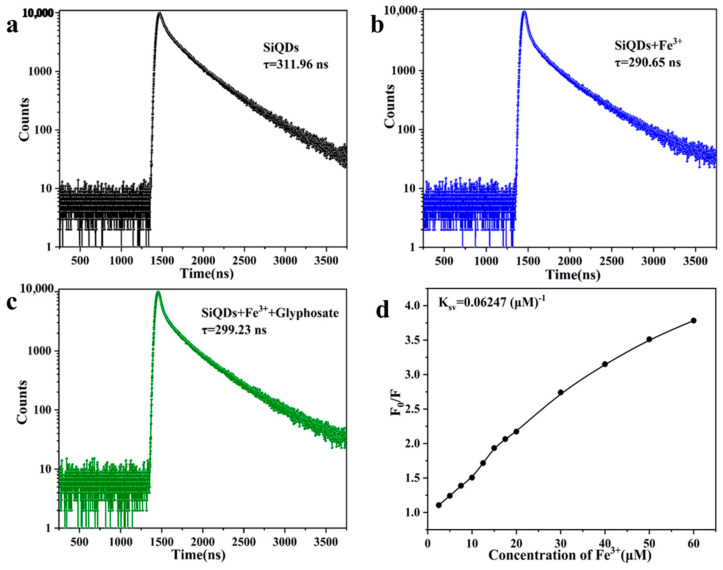
Fluorescence decay curve and corresponding fluorescence lifetime of (**a**) SiQDs; (**b**) SiQDs, Fe^3+^; (**c**) SiQDs, Fe^3+^ and glyphosate; (**d**) Stern-Volmer plot based on fluorescence intensity ratio (F/F_0_) versus Fe^3+^ concentration.

The fluorescence mechanisms of “Turn-off” and “Turn-on” in the SiQDs/Fe^3+^ system were investigated by time-resolved fluorescence lifetime measurements of SiQDs alone, in the presence of Fe^3+^, and in the presence of Fe^3+^ and glyphosate in the SiQDs aqueous solution. Figure 11a shows that the fluorescence lifetime of SiQDs alone was 311.96 ns; when Fe^3+^ was added to the SiQDs solution, the fluorescence lifetime was reduced to 290.65 ns (Figure 11b), which suggests that the addition of Fe^3+^ to the SiQDs solution resulted in the coordination of Fe^3+^ with the -OH and other groups on the surface of SiQDs [18,19]. When glyphosate was added, the fluorescence lifetime became 299.23 ns (Figure 11c). It has been shown that the phosphonyl (-PO_3_H_2_) and carboxyl (-COOH) groups in the glyphosate molecule are capable of complexing with Fe^3+^ [20,21]. Based on this, we hypothesized that when glyphosate was added to the mixed solution of SiQDs and Fe^3+^, the complexation with Fe^3+^ was stronger than the coordination between Fe^3+^ and groups such as -OH on the surface of SiQDs, which ultimately led to a certain degree of fluorescence recovery of SiQDs.

In addition, the Stern-Volmer equation can be used to describe the relationship between the fluorescence intensity of SiQDs and the concentration of the quenching agent.

(2) $$\frac{F_{0}}{F}=1+K_{SV}\left[Q\right]=1+K_{q}\tau_{0}\left[Q\right]$$

In Equation (2), F and F_0_ are the fluorescence intensities of SiQDs in the presence and absence of Fe^3+^, [Q] is the concentration of the quencher Fe^3+^, K is the Stern-Volmer bursting constant, and τ_0_ is the initial fluorescence decay lifetime.

Typically, a static burst is recognized to occur when Kq is greater than 2 × 10^10^ L mol^−1^s^−1^ [28]. Calculated from Figure 11d, Kq = 2 × 10^11^ L mol^−1^s^−1^, whereas the fluorescence lifetimes did not change much before and after the addition of Fe^3+^, as shown in Figure 11a,b. Therefore, it is hypothesized that the fluorescence quenching mechanism is ascribed to static quenching.

**Figure 12:**
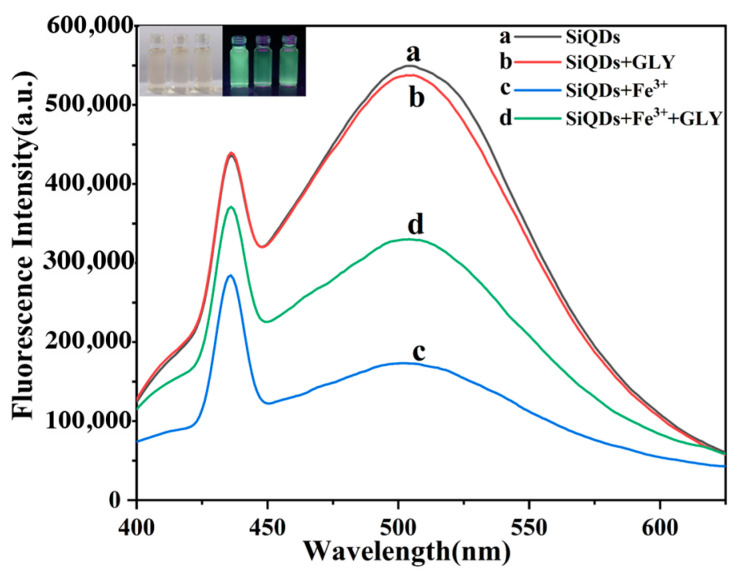
Fluorescence spectra of (a) SiQDs; (b) SiQDs and glyphosate; (c) SiQDs and Fe^3+^; (d) SiQDs, Fe^3+^ and glyphosate. The insets show photographs of SiQDs, the SiQDs/Fe^3+^ system and SiQDs/Fe^3+^ solution after reaction with glyphosate under natural light and 365 nm UV light.

Based on the above speculations, we investigated the changes in the fluorescence spectra of the mixed solutions after adding Fe^3+^, glyphosate, and both Fe^3+^ and glyphosate to the aqueous solution of SiQDs, respectively, and the specific experimental results are shown in Figure 12. As can be seen by analyzing the figure, when glyphosate alone was added to the aqueous solution of SiQDs, the fluorescence intensity basically remained stable without significant changes, which leads to the conclusion that glyphosate does not interact directly with SiQDs. When a certain concentration of Fe^3+^ was introduced into the aqueous solution of SiQDs alone, the fluorescence intensity of the system showed an obvious burst phenomenon. Subsequently, with further addition of glyphosate to the Fe^3+^ and SiQDs system, the fluorescence intensity showed an obvious recovery trend, which is consistent with the change in the phenomenon in the upper left inset. The inset of Figure 12 shows solutions of SiQDs under daylight and 365 nm UV lamp irradiation (from left to right, SiQDs alone, SiQDs/Fe^3+^ system, and SiQDs/Fe^3+^ after reaction with glyphosate), and it can be clearly seen that SiQDs are a light orange liquid under daylight, and an aqueous solution of SiQDs under irradiation of a 365 nm UV lamp showed a strong green fluorescence, which was strong enough to be observed with the naked eye. When Fe^3+^ was added, the fluorescence burst phenomenon occurred; the intensity was reduced, and then the fluorescence was restored, and green fluorescence was emitted when glyphosate was added.

In summary, glyphosate has a specific effect on the fluorescence of SiQDs in the SiQDs/Fe^3+^ system, which is reflected in the recovery of fluorescence of SiQDs after fluorescence extinction by Fe^3+^.

### 3.5. Detection of Glyphosate in Real Samples

Recovery experiments were conducted to evaluate the usefulness of the method. The experimental samples were selected from potatoes and yams purchased from supermarkets. A series of glyphosate standard solutions with final concentrations of 6 μg/mL, 8 μg/mL and 10 μg/mL were added to the treated real samples for fluorescence determination, and the recoveries of glyphosate were calculated.

From the table, it can be seen that the recoveries of both samples were 91.69–104.53% with RSD below 2.0%. The results are listed in Table 1. It shows that the fluorescence detection system is reliable for the detection of glyphosate pesticide residues in real samples.

## 4. Conclusions

In summary, this work develops a SiQDs/Fe^3+^ fluorescent probe to quantitatively detect and analyze glyphosate residues based on the response mechanism that Fe^3+^ can statically quench the fluorescence of SiQDs and the functional groups in glyphosate complex with Fe^3+^, which allows the fluorescence of SiQDs to recover. The SiQDs prepared by the facile room-temperature stirring approach were well-dispersed spherical particles with an average size of 3.04 nm under TEM. Characterization analysis using FTIR spectroscopy, XPS, and XRD showed that the SiQDs in an amorphous state were successfully prepared with elements of Si, C, O, and N on their surfaces, and the fluorescence quantum yield was 13.97%. Under the optimal detection conditions, the constructed SiQDs/Fe^3+^ fluorescent probe exhibited satisfactory reproducibility and excellent anti-interference ability and was successfully applied to real sample analysis. Based on the rational design of the SiQDs/Fe^3+^ fluorescence nanosensor, this study provides a novel strategy for the detection of glyphosate residues.

## Figures and Tables

**Table 1:** Recovery experiments of glyphosate detection in real samples.

Sample	Added (μg/mL)	Found (μg/mL)	Recovery (%)	RSD (%)
Potato	6	5.50	91.69	0.51
	8	8.36	104.53	1.23
	10	10.06	100.63	1.09
Yam	6	5.68	94.61	0.94
	8	7.60	95.02	1.14
	10	9.76	97.64	0.48

## Data Availability

Data is contained within the article and Supplementary Materials Table S1.

## Supplementary Materials

The following supporting information can be downloaded at: https://www.mdpi.com/article/10.3390/s26092851/s1, Table S1: Comparison of various glyphosate probes [29,30,31,32,33,34,35,36,37].
