# Supplementary material for: Selective Fluorescence Detection of Glyphosate Pesticide Residue Based on Fe3+ Modulated SiQDs Nanosensors
Source: Sensors (Basel). 2026 May 2;26(9):2851. doi: 10.3390/s26092851 (PMC13165916; doi:10.3390/s26092851)
Supplement: Supplementary file 1 [file sensors-26-02851-s001.zip › sensors-4261263-supplementary.pdf]

Supporting Information for:

Selective fluorescence detection of glyphosate pesticide residue based on Fe^{3+} modulated SiQDs nanosensors

Ruonan Li,[a, b] Jian Xu [a,*] and Fankui Zeng [a,*]

[a] Research Center for Natural Medicine and Chemical Metrology, Lanzhou Institute of Chemical Physics, Chinese Academy of Sciences, Lanzhou 730000, P. R. China.

[b] University of Chinese Academy of Sciences, Beijing, 100049, China.

[*]Corresponding author, E-mail: xujian1980@licp.cas.cn; zengfk@licp.cas.cn

Table S1

Comparison of various glyphosate probes.

**Method/ probe**	**Response time (min)**	**Detection limit (μM)**	**Reference**
Coumarin derivative/Cu^{2+}	5	0.11	*Anal. Methods,* 2020, 12, 520.
AuNPs/Pb^{2+}	15	2.4×10^{-3}	*Anal. Methods,* 2017, 9, 2890.
DNA-AgNCs/Cu^{2+}	1	3×10^{-2}	*Food Chem,* 2022, 367, 130617.
Rhodamine B/AuNPs	5	5.9×10^{-4}	*Anal. Chem.,* 2012, 84, 4185.
AuNPs/Cys	15	5.9	*Analyst,* 2019, 144, 2017.
Rhodamine/Cu^{2+}	2	4.1×10^{-3}	*Talanta,* 2021, 224, 121834.
CDs/Cu^{2+}	12	0.095	*RSC Adv,* 2016, 6, 85820.
lgG-CDs	120	0.047	*J. Agric. Food. Chem.,* 2016, 64, 6042.
GMP/Tb@GMP/Eu/DPA	30	41	*Food Chem,* 2020, 323, 126815.
SiQDs/Fe^{3+}	5	2.3×10^{-3}	this work
